# Serum NfL and GFAP as biomarkers of progressive neurodegeneration in TBI

**DOI:** 10.1002/alz.13898

**Published:** 2024-05-28

**Authors:** Pashtun Shahim, Dzung L. Pham, Andre J. van der Merwe, Brian Moore, Yi‐Yu Chou, Sara M. Lippa, Kimbra Kenney, Ramon Diaz‐Arrastia, Leighton Chan

**Affiliations:** ^1^ Rehabilitation Medicine Department National Institutes of Health (NIH) Clinical Center Bethesda Maryland USA; ^2^ National Institutes of Neurological Disorders and Stroke, NIH Bethesda Maryland USA; ^3^ Department of Neurology MedStar Georgetown University Hospital, Pasquerilla Healthcare Center Washington District of Columbia USA; ^4^ The Military Traumatic Brain Injury Initiative (MTBI2) Bethesda Maryland USA; ^5^ The Henry M. Jackson Foundation for the Advancement of Military Medicine Bethesda Maryland USA; ^6^ Uniformed Services University of the Health Sciences Bethesda Maryland USA; ^7^ National Intrepid Center of Excellence, Walter Reed National Military Medical Center Bethesda Maryland USA; ^8^ Department of Neurology University of Pennsylvania Perelman School of Medicine Philadelphia Pennsylvania USA

**Keywords:** brain volume, glial fibrillary acidic protein, neurofilament light, tau, traumatic brain injury, ubiquitin C‐terminal hydrolase‐L1

## Abstract

**BACKGROUND:**

We examined spatial patterns of brain atrophy after mild, moderate, and severe traumatic brain injury (TBI), the relationship between progression of brain atrophy with initial traumatic axonal injury (TAI), cognitive outcome, and with serum biomarkers of brain injury.

**METHODS:**

A total of 143 patients with TBI and 43 controls were studied cross‐sectionally and longitudinally up to 5 years with multiple assessments, which included brain magnetic resonance imaging, cognitive testing, and serum biomarkers.

**RESULTS:**

TBI patients showed progressive volume loss regardless of injury severity over several years, and TAI was independently associated with accelerated brain atrophy. Cognitive performance improved over time. Higher baseline serum neurofilament light (NfL) and glial fibrillary acidic protein (GFAP) were associated with greater rate of brain atrophy over 5 years.

**DISCUSSSION:**

Spatial patterns of atrophy differ by injury severity and TAI is associated with the progression of brain atrophy. Serum NfL and GFAP show promise as non‐invasive prognostic biomarkers of progressive neurodegeneration in TBI.

**Highlights:**

- In this longitudinal study of patient with mild, moderate, and severe traumatic brain injury (TBI) who were assessed with paired magnetic resonance imaging (MRI), blood biomarkers, and cognitive assessments, we found that brain atrophy after TBI is progressive and continues for many years even after a mild head trauma without signs of brain injury on conventional MRI.
- We found that spatial pattern of brain atrophy differs between mild, moderate, and severe TBI, where in patients with mild TBI , atrophy is mainly seen in the gray matter, while in those with moderate to severe brain injury atrophy is predominantly seen in the subcortical gray matter and whiter matter.
- Cognitive performance improves over time after a TBI.
- Serum measures of neurofilament light or glial fibrillary acidic protein are associated with progression of brain atrophy after TBI.

## 1 BACKGROUND

Traumatic brain injury (TBI) is recognized as a risk factor for late‐life neurodegeneration.^1^, ^2^, ^3^, ^4^, ^5^, ^6^, ^7^ Brain atrophy is consistently observed in the months to years after TBI.^1^, ^2^, ^3^, ^4^, ^5^ Brain atrophy can be quantified using volumetric magnetic resonance imaging (MRI), either with voxel‐based morphometry,^8^ or summary measures from regions of interest.^9^, ^10^ Most current MRI studies have investigated brain atrophy in acute TBI, subacute TBI, or within a year after moderate or severe TBI, which are confounded by the acute effects of injury, including cerebral edema.^11^, ^12^ Furthermore, several of the previous MRI brain volume studies had small sample sizes or acquisition on different scanners, making the interpretation of subject variability difficult.^13^, ^14^, ^15^, ^16^, ^17^, ^18^ Additionally, there are few studies of brain volumes in individuals who have sustained a mild TBI,^19^ with the largest study only including seven patients who were scanned 3 months apart.^13^ Determining the time course of atrophy after mild, moderate, and severe TBI in the chronic phase is relevant for several reasons, including evaluation of the long‐term effects of neuroprotective interventions.

TBI is associated with cognitive impairment, affecting several key domains, including attention, executive function/processing speed, language, and memory.^20^ Furthermore, patients with TBI often report reduced quality of life (QoL).^21^ While several studies report cognitive impairment and reduced QoL after TBI, it is currently unclear whether the cognitive decline continues over time. A detailed characterization of the relationship between long‐term changes in cognitive outcomes and MRI measures of brain atrophy in adequately powered cohorts is needed to inform the design of future clinical trials of neuroprotective or neurorestorative therapies.

While MRI volumetric analysis can sensitively quantify brain tissue loss, recent advances in the ultrasensitive immunoassay technology field have made it possible to quantify neuronally derived proteins in peripheral blood reliably and rapidly.^22^, ^23^ In particular, axonal proteins such as neurofilament light (NfL) and tau have emerged as potential markers of neurodegeneration.^22^, ^23^, ^24^ Highly sensitive immunoassays for glial fibrillary acidic protein (GFAP), a marker of astrogliosis, and ubiquitin C‐terminal hydrolase‐L1 (UCH‐L1), a cytosolic neuronal protein, have also received much attention as markers of neuronal injury or death.^25^, ^26^, ^27^ While these biomarkers have been extensively assessed in other neurodegenerative diseases,^28^ and in acute or subacute TBI,^29^ their relationship to changes in global and regional brain volumes and cognitive performance in the months to years after TBI has yet to be fully examined.

In previous reports, we assessed the associations among serum NfL, GFAP, tau, and UCH‐L1 and global measures of MRI‐measured brain atrophy.^30^, ^31^ In this study, we examined spatial patterns of brain atrophy after mild, moderate, and severe TBI, the relationship between progression of brain atrophy with baseline diffusion tensor imaging (DTI) estimates of traumatic axonal injury (TAI), cognitive outcomes, and with serum biomarkers of neuronal injury and astrogliosis. We specifically assessed cross‐sectional and longitudinal differences in gray and white matter volumes over 5 years, hypothesizing that TBI would result in progressive brain atrophy, and the rate of brain atrophy would correlate with TBI severity and DTI estimates of TAI. We also assessed whether these changes relate to changes in cognitive outcomes and serum biomarkers, hypothesizing that changes in these measures would follow the trajectory of brain volume over time and that serum biomarkers at baseline would predict future changes in brain atrophy and cognitive outcomes.

## 2 METHODS

### 2.1 Study design and participants

A detailed study description including the inclusion and exclusion criteria can be found on ClinicalTrials.gov (Identifier: NCT01132898) and in Appendix S1 in supporting information. The severity of TBI was based on the Department of Defense and Veteran Affairs (DoD/VA) criteria^32^ and clinical history. Participants were enrolled between January 2011 and February 2020 at the National Institutes of Health (NIH) Clinical Center, Bethesda, Maryland, USA. For the longitudinal part of the study, we enrolled patients who had suffered a TBI within the past year and followed them over 5 years. The participants were also offered longitudinal blood, imaging, and outcome assessments at 30 (± 10 days), 90 (± 30 days), and 180 days (± 30 days), and at 1, 2, 3, 4, and 5 years (± 2 months). This study also had a cross‐sectional part, in which we enrolled those who had suffered a TBI within the past 5 years.

### 2.2 Blood, clinical, and imaging outcomes

The main outcome measures were changes in global and regional brain volumes from 30 days to 5 years after TBI in relation to TBI diagnosis; injury severity; cognitive composite scores; QoL; and serum concentrations of NfL, tau, GFAP, and UCH‐L1. We also assessed whether the presence of TAI is associated with the progression of neurodegeneration or brain atrophy. TAI is characterized by microhemorrhages within white matter (WM),^33^ which can be detected using gradient echo and susceptibility weighted imaging; however, these techniques do not provide a quantitative assessment of WM integrity and patients without microhemorrhage may still show evidence of TAI.^34^ We therefore used DTI, which provides quantitative information about WM damage. The corpus callosum (CC) is a central WM structure within the brain that is highly susceptible to TAI and may be used as a surrogate marker for TAI.^35^ We quantified TAI in the CC using DTI fractional anisotropy (FA), axial diffusivity (AD), radial diffusivity (RD), and mean diffusivity (MD). We hypothesized that FA would be reduced, and that AD, RD, and MD would be increased in the chronic phase of TAI.^36^

### 2.3 Image acquisition and processing

High resolution structural MR and diffusion weighted images (DWIs) were acquired on a 3 Tesla MR scanner (Siemens Biograph) with a 16‐channel head coil in Radiology and Imaging Sciences at the NIH, Bethesda, Maryland, USA. Structural images were acquired on the same scanner as DWIs. All participants were scanned on the same scanner. The imaging parameters and methodology are described in detail in Appendix S1.

**RESEARCH IN CONTEXT**

1. **Systematic review**: Previous magnetic resonance imaging (MRI) studies have investigated brain atrophy in acute traumatic brain injury (TBI), subacute TBI, or within a year after moderate or severe TBI in a small number of individuals or with acquisition on different MRI scanners, making the interpretation of subject variability difficult. We searched PubMed and found no studies that have assessed brain atrophy after mild, moderate, or severe TBI over several years. Furthermore, no previous studies have conducted a detailed longitudinal assessment of cognitive performance after TBI. Last, the relationships among brain atrophy, cognitive performance, and serum biomarkers of brain injury after TBI have not been assessed in detail previously.
2. **Interpretation**: We show that brain atrophy after TBI is progressive and continues for many years even after a mild head trauma without signs of brain injury on conventional MRI. In contrast, cognitive performance improves over the same period, suggestive of two separate processes. Serum measures of neurofilament light (NfL) or glial fibrillary acidic protein (GFAP) are associated with progression of brain atrophy after TBI.
3. **Future directions**: Our findings suggest that a single TBI triggers a progressive neurodegeneration that continues for many years. Serum NfL and GFAP can be used as non‐invasive prognostic biomarkers of progressive neurodegeneration in TBI.

### 2.4 Neuropsychological assessments

We constructed composites scores for five key cognitive domains (attention/processing speed, executive functioning, language, delayed memory, and working memory), where each comprised of several neuropsychological tests. The neuropsychological tests are detailed in the Appendix S1. In addition to the cognitive domains, we assessed QoL, using the Satisfaction with Life Scale (SWLS).^37^

### 2.5 Blood handling and processing

Serum NfL, GFAP, tau, and UCH‐L1 concentrations were measured using the Neurology 4‐plex assay kit (Quanterix Corporation) on a single molecule array HD‐1 Analyzer (Quanterix Corporation). The methodology is detailed in Appendix S1. All samples were processed and analyzed at NIH, Bethesda, MD, USA, using the same batch of reagents by certified laboratory technicians blinded to clinical information.

### 2.6 Statistical analysis

We conducted four specific analyses to assess the baseline and longitudinal relationships among the neuroimaging, cognitive, and serum biomarker outcomes. First, we constructed linear mixed effects (LME) models with either longitudinal brain volumes, or cognitive composite scores as the dependent variable, and age, sex, and education as independent variables. We adjusted for these demographic factors given their correlations with brain atrophy or neurodegeneration, and we included education given its potential impact on recovery.^38^ The average changes in brain volumes in percentage per year (for ease of interpretability) were calculated by dividing the slope from the LME models with the intercept for each group and brain region. In a subset of controls with two MRI scans, we calculated the annualized rate of brain atrophy as follows: 100× (follow‐up volume − baseline volume/baseline volume)/interval years. This method was preferred to LME due to the availability of only two time points and the regions of interest were adjusted to estimated total intracranial volume. Second, we tested the relationship between TAI and brain atrophy by assessing the correlation between the slope from the LME models with baseline DTI estimates of TAI. Third, we tested the cross‐sectional relationships among brain volumes, serum biomarkers, and neuropsychological tests using linear regression, covaried for age, sex, education, and time since most recent TBI, followed by correction for multiple comparisons using the Benjamini–Hochberg method. Fourth, we tested correlations between longitudinal brain volumes (in all the brain regions from LME models that survived multiple comparison testing) and longitudinal data for cognitive composite scores, and serum biomarkers. We computed Spearman rank correlations between individual‐specific random slope from LME models for brain volumes and random slopes for neuropsychological measures, and serum biomarkers. Biomarker concentrations in serum were non‐normally distributed because of biological plausibly higher values. Natural log‐transformation produced plausibly normal distributions and was used for all correlation analyses. All LME models included random intercepts and were adjusted for age, education, and sex. Time was treated as a continuous variable. The models were fit using maximum likelihood estimation and differences in trajectories (e.g., estimated change or slope) across groups were assessed using approximate *F* or *t* tests. We used the Benjamini–Hochberg test correcting for multiple comparisons when appropriate. The specific statistical method including multiple comparisons is also detailed in the figure or table legends. All statistical analyses were performed in R (v.3.0.3, The R Foundation for Statistical Computing).

### 2.7 Standard protocol approvals, registrations, and patient consents

The study was approved by the NIH Institutional Review Board. All participants gave written and informed consent.

## 3 RESULTS

A total of a total of 218 participants (175 with TBI [median, 0.7 years, interquartile range (IQR) 0.2–1.4 years since most recent TBI], and 43 healthy controls) were enrolled between January 2011 and February 2020. Blood, MRI, and cognitive outcome data were available on 143 participants with TBI at the time of the analysis, of whom 80 were classified as mild TBI and had no abnormalities on conventional MRI, 41 were moderate, and 22 were severe. Of 143 TBI participants, 82 of the participants had two or more follow‐up visits with paired MRI, blood, and outcome assessment, while 54 participants had four or more follow‐up visits with paired MRI, blood, and outcome assessment. The average interimaging interval for the patients in the overall analysis was 3.0 years (median 3.1 years, IQR 1.0–4.6). The imaging, blood, and outcome assessments were performed within 1 day of the MRI. Twelve control participants (median age 47 years, IQR 42–53) underwent a follow‐up MRI at an average of 7 months (Table S1 in supporting information S1). The demographic and clinical characteristics of the participants at baseline are shown in Table 1. Age, sex, race, and education did not differ significantly between the TBI and control groups (Table 1).

**TABLE 1 alz13898-tbl-0001:** Demographic and clinical characteristics of TBI patients and controls at baseline.

Characteristics	TBI	Controls	*P* value
*n*	143	39	−
Age, years, mean ± SD	43 (16)	42 (12)	0.67
Sex, female/male	51/92	15/24	0.75
Race, no. (%)			0.75
White	105 (73)	24 (61)	
Black	20 (14)	13 (33)	
Asian	4 (3)	1 (3)	
Multiple races	10 (7)	1 (3)	
Other	4 (3)	0 (0.0)	
Years of education, mean ± SD	16 (3)	17 (3)	0.11
Time since most recent TBI, year, median (IQR)	0.7 (0.2–1.4)	−	−
Loss of consciousness, no. (%)			
Yes	76 (53)	−	
No	67 (47)	−	
Posttraumatic amnesia, no. (%)			
Yes	63 (44)	−	
No	80 (56)	−	
Injury severity, no. (%)			
Mild^a^	80 (56)	−	−
Moderate	41 (29)	−	−
Severe	22 (15)	−	−
Cause of injury, no. (%)			
Acceleration/deceleration	47 (28)	−	−
Blast	8 (5)	−	−
Direct impact‐blow to the head	46 (28)	−	−
Fall	50 (30)	−	−
Other	11 (9)	−	−
Cognitive composite scores, mean (SD)			
*n*	100	18	
Attention/processing speed	49 (10)	52 (7)	0.21
*n*	71	18	
Executive functioning	46 (8)	51 (7)	0.014
*n*	71	18	
Language composite	44 (9)	49 (10)	0.06
*n*	93	18	
Delayed memory	48 (15)	55 (9)	0.05
*n*	96	18	
Working memory	53 (10)	54 (9)	0.64
Quality of life, *n*	103	19	
Satisfaction with Life Scale	23 (7)	29 (5)	0.0003

*Notes*: Differences between categorical variables were calculated using chi^2^ tests. Differences between continuous variables were calculated using *t* tests. Cognitive composite scores are presented as *t* scores (50 is population mean, +/− 10 is one standard deviation greater or lower).

Abbreviations: CT, computed tomography; IQR, interquartile range; MRI, magnetic resonance imaging; SD, standard deviation; TBI, traumatic brain injury.

^a^ Patients with mild TBI had no abnormalities intracranial abnormalities on CT head or conventional MRI sequences.

### 3.1 Lower cross‐sectional brain volumes in patients with TBI at baseline

**FIGURE 1 alz13898-fig-0001:**
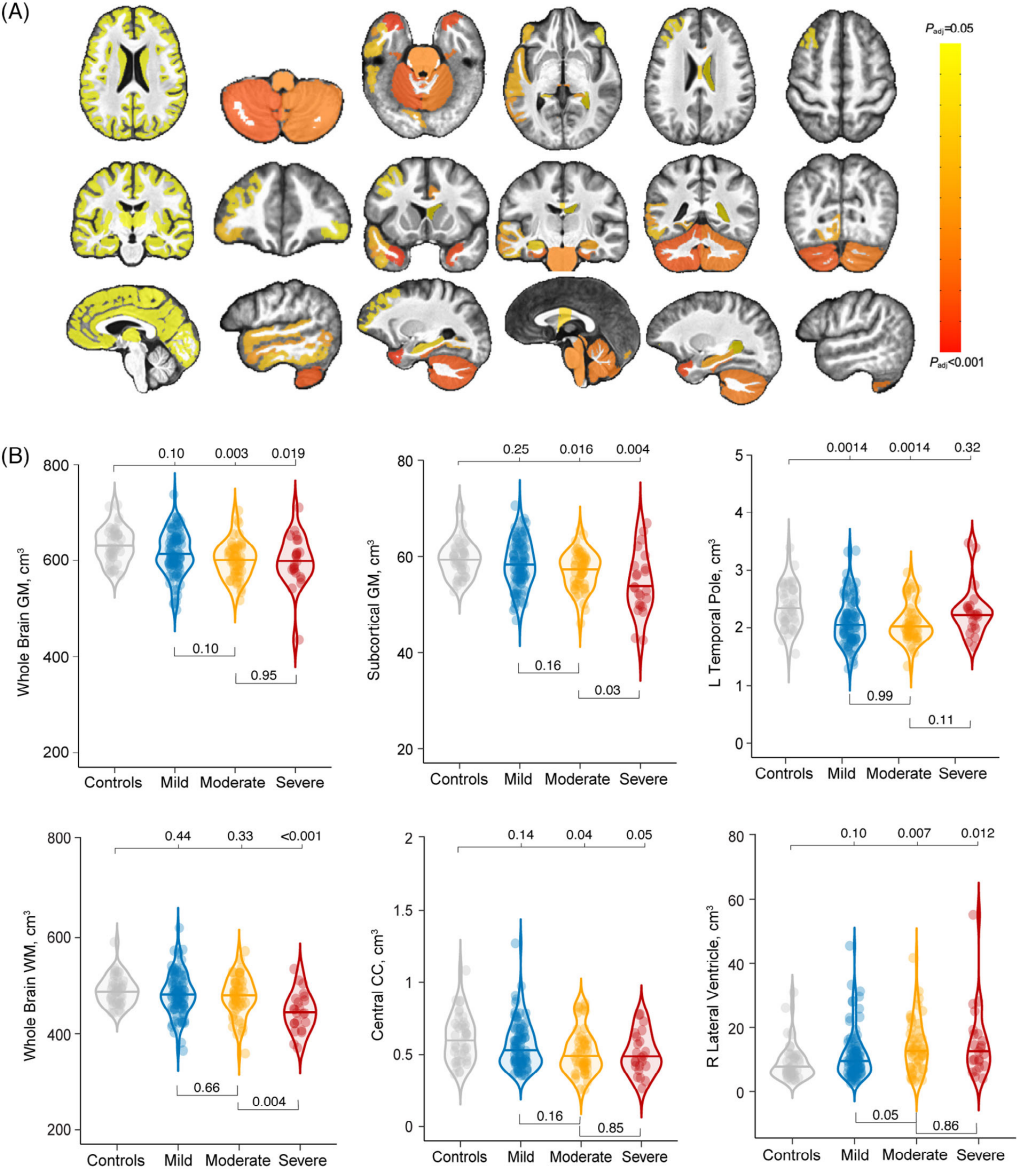
Lower cross‐sectional brain volumes in patients with TBI at baseline. A, There are significantly lower brain volumes in TBI patients compared to controls at baseline, corrected for multiple comparisons using the Benjamini–Hochberg procedure. The T_1_‐MR structural slices displayed are axial, coronal, and sagittal and overlaid on average of 15 participants. B, Volume differences in key brain regions are associated with brain atrophy in patients with a history of mild, moderate, and severe TBI, as well as controls. CC, corpus callosum; GM, gray matter, L, left; MR, magnetic resonance; R, right; TBI, traumatic brain injury; WM, white matter.

At baseline,[Table alz13898-tbl-0001] TBI patients (all severities) had multiple regions of significantly lower gray matter (GM) and WM brain volumes compared to controls (Figure 1A). For GM, it included the frontal, temporal, cerebellar, parahippocampal, and hippocampal regions. For WM, it included multiple regions, including the brainstem, the CC, and lateral ventricles. There were no brain regions with significantly greater volume in TBI patients compared to controls. The differences in volume loss between left and right brain hemispheres were not assessed.

Assessing the volume differences across TBI severity, patients with severe TBI had lower brain volumes than moderate cases across all the above‐mentioned regions; however, the differences were not statistically significant after correcting for multiple comparisons, except for greater atrophy in the whole brain WM (WBWM; Figure 1B). Comparing mild TBI versus controls, those with mild TBI had significantly lower brain volumes in the frontal (rostral and caudal anterior cingulate cortex) and temporal (bilateral temporal poles, and parahippocampal) regions, and the cerebellar cortex (Figure 1B).

### 3.2 Longitudinal brain volume reduction in patients with TBI

**FIGURE 2 alz13898-fig-0002:**
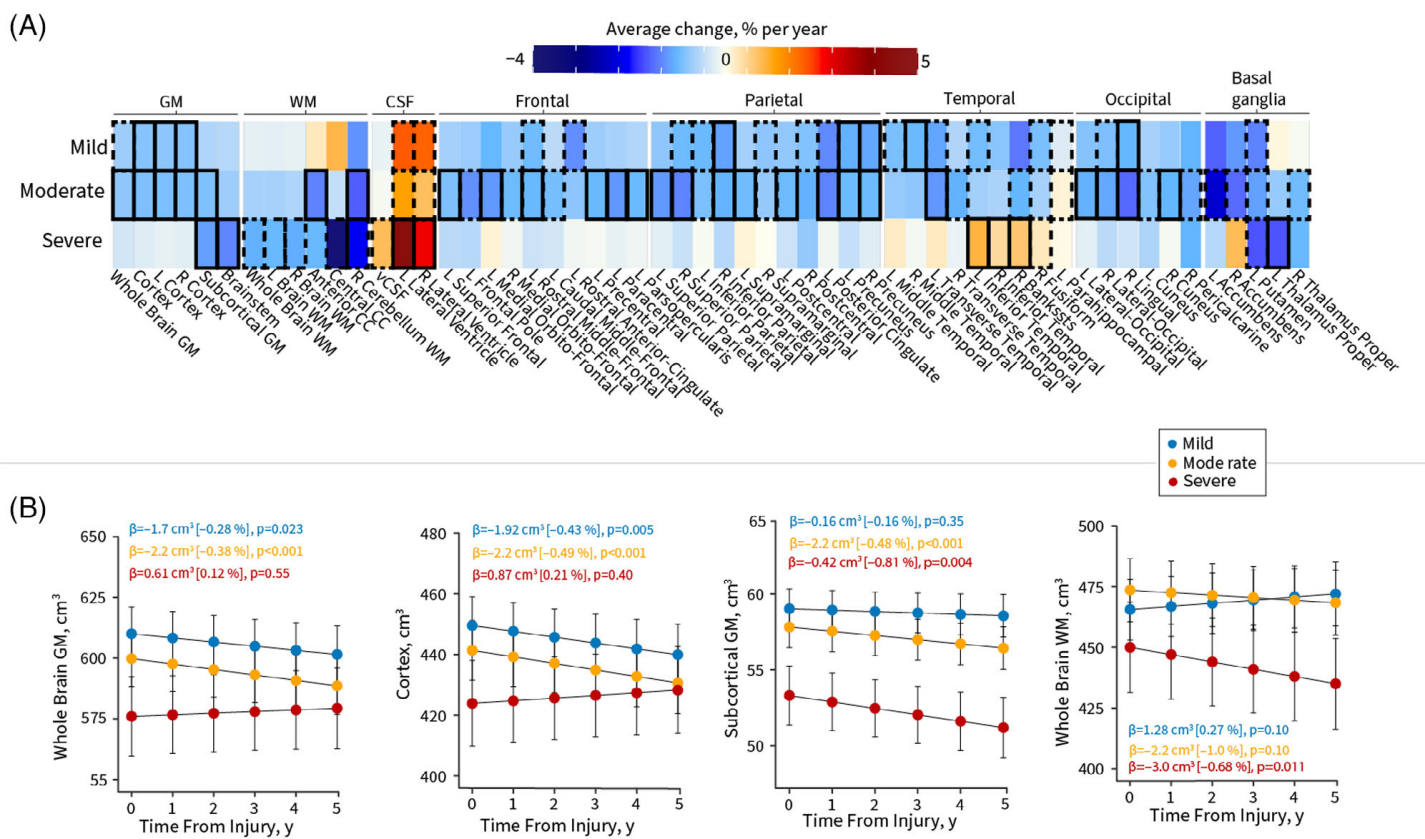
Longitudinal changes in brain volume. A, Longitudinal changes in brain volumes calculated as average change in percentage per year in patients with a history of mild, moderate, or severe TBI. The average changes in brain volumes in percentage per year (for ease of interpretability) were calculated by dividing the slope from the linear mixed‐effects model with the intercept for each group and brain region. Bold outlines, *P *< 0.01; dashed outlines *P *< 0.05, Benjamini–Hochberg corrected. Cooler colors indicate decreases in brain volumes and hotter colors indicate increases in brain volumes. B, Examples of longitudinal changes in brain volume across mild, moderate, and severe TBI are shown in the heatmap. The fitted lines indicate mean, and standard errors are from the linear mixed‐effects models covaried for age, education, and sex. The trajectory of brain volume changes at individual level for key brain regions are shown in Figure S1 in supporting information. bankssts, bank of the superior temporal sulcus; CC, corpus callosum; GM, gray matter; L, left; R, right; vCSF, ventricular cerebrospinal fluid; WM, white matter.

Figure 2 shows the average yearly changes in brain volume across mild, moderate, and severe TBI patients, independent of age, sex, and education. The trajectory of brain volume changes at individual participant level for key brain regions are shown in Figure S1 in supporting information. Over the course of 5 years, patients with mild TBI lost an average of 0.43% of cortex volume, 0.28% of whole brain GM volume (WBGM), 0.16% of subcortical GM volume, and gained 0.27% of WBWM volume and 0.31% in ventricle cerebrospinal fluid (CSF) volumes per year, respectively (Figure 2A). Over the same period, patients with moderate TBI lost an average of 0.49% of cortex volume, 0.38% of WBGM volume, 0.48% of subcortical GM, 1.0% of WBWM volume, and gained 0.41% ventricle CSF volumes per year. Patients with severe TBI lost an average of 0.12% of WBGM, 0.81% of subcortical GM volume, 0.68% of WBWM volume per year, and gained an average of 0.21% of cortex and 1.32% in ventricular CSF volumes, respectively.

As indicated, the spatial pattern of atrophy differed between mild and moderate‐to‐severe TBI. In those with mild TBI, decreases were seen in the WBGM, cortex, frontal, temporal, parietal, and occipital lobes, while volumes increased in the lateral ventricles (Figure 2B). Patients with moderate TBI showed significant decline in volume in several GM, subcortical GM, and WM regions, while increases were seen in ventricle volumes (Figure 2A). In contrast, in patients with severe TBI the decrease in brain volume was more pronounced in WBWM, subcortical GM, and basal ganglia, while increases were seen in ventricle volumes (Figure 2A). Increases were also observed in frontal and inferior temporal volumes for those with severe TBI (Figure 2A).

Longitudinally, controls lost on average 0.52% (95% confidence interval [CI], −2.7 to 1.6) of WBGM, 0.30% (95% CI, −3.4 to 1.6) of subcortical GM, and gained 0.45% (95% CI, −1.6 to 2.5) in WBWM per year.

### 3.3 TAI is associated with the progression of brain atrophy

**FIGURE 3 alz13898-fig-0003:**
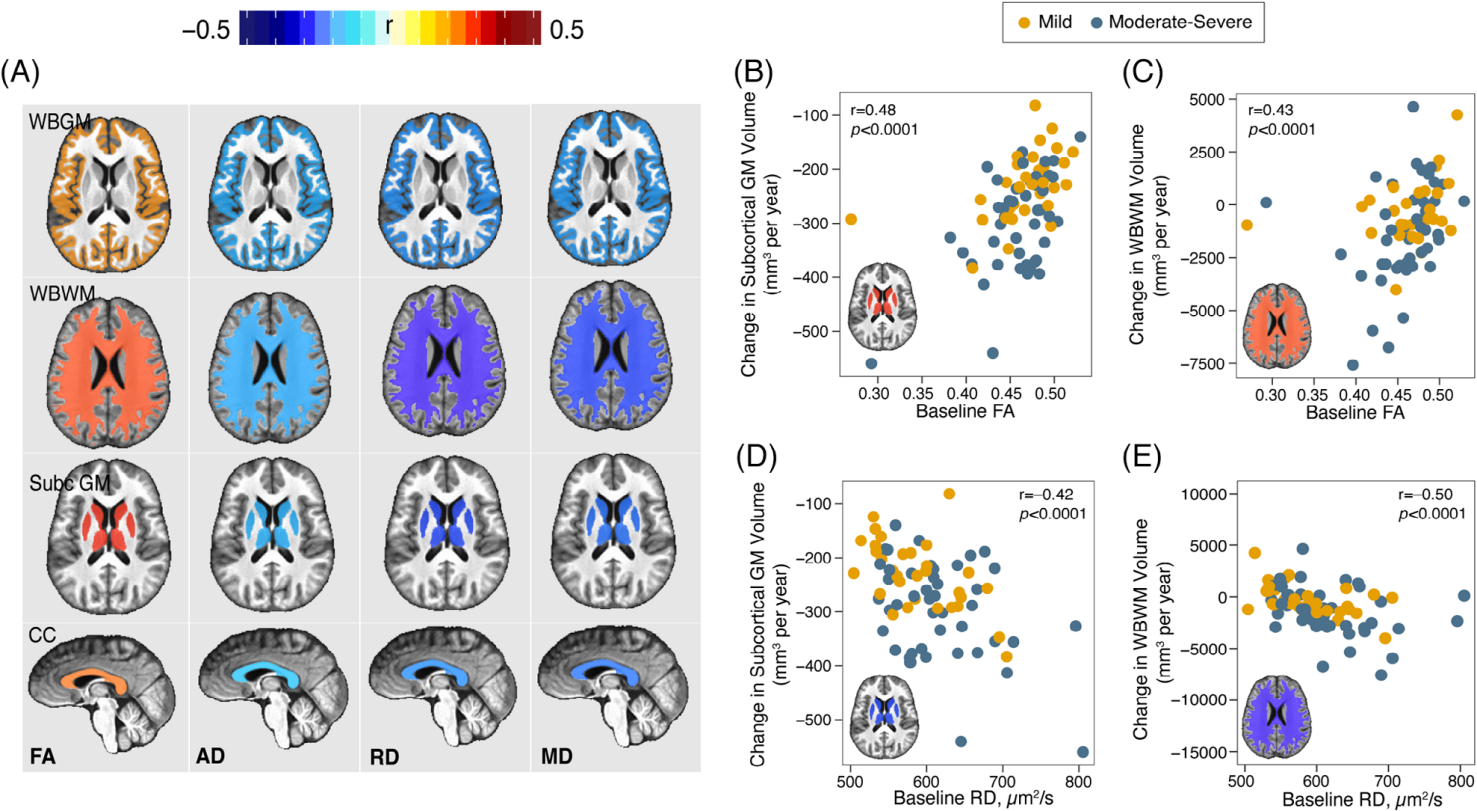
TAI underlies the progression of brain atrophy. A, Correlations between DTI measures of TAI (FA, AD, RD, and MD for CC) at baseline and the rate of change (per year) in MRI‐measured brain atrophy (GM, WM, subcortical GM, and CC volumes). The cooler colors in the heatmap indicate negative correlation and the hotter colors indicate positive. The colors of the brain masks (whole brain GM, whole brain WM, subcortical GM, and CC volumes) in panel (A) correspond to the correlation (*r*) between various TAI measures (FA, AD, RD, and MD) and atrophy in the WBGM, WBWM, subcortical GM, and CC volumes. B–E, Examples of the correlations summarized in the panel (A). Changes in MRI‐measured atrophy were tested using mixed‐effects models covaried for age, sex, and education. The association between the individual slope from the mixed‐effects model and the baseline biomarker concentrations were tested using Spearman rank correlation (*r*). All the associations shown in the heatmap were statistically significant. AD, axial diffusivity; CC, corpus callosum; DTI, diffusion tensor imaging; FA, fractional anisotropy; GM, gray matter; MD, mean diffusivity; MRI, magnetic resonance imaging; RD, radial diffusivity; TAI, traumatic axonal injury; WBGM, whole brain gray matter; WBWM, whole brain white matter; WM, white matter.

We found that decreased FA and increased AD, RD, and MD of the CC at baseline were significantly associated with accelerated MRI‐measured brain atrophy (Figure 3). These effects were seen independent of age, sex, and level of education.

### 3.4 Longitudinal changes in cognitive composite scores

**FIGURE 4 alz13898-fig-0004:**
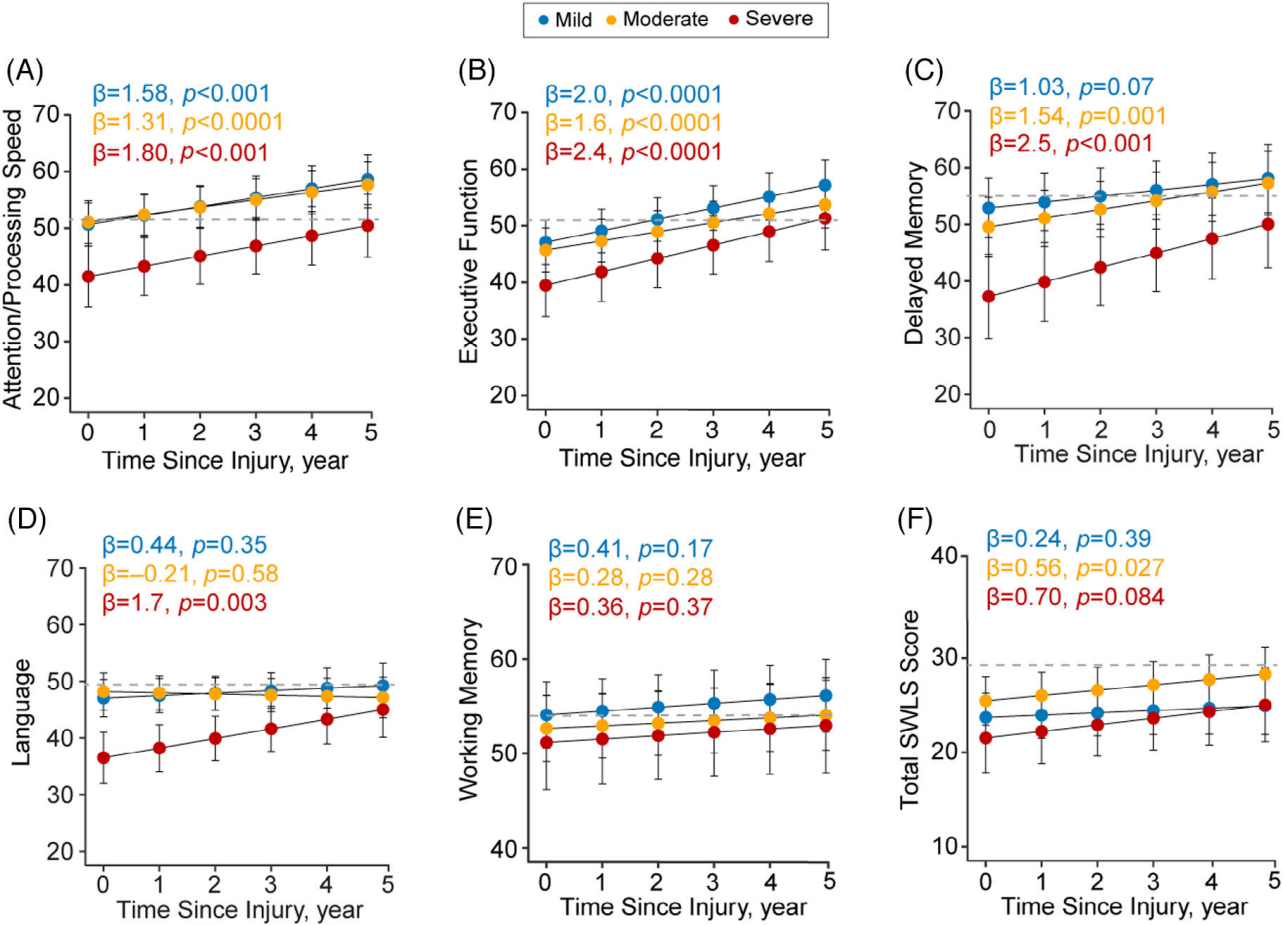
Longitudinal changes in cognitive composite scores and quality of life. A–F, Longitudinal changes in cognitive composite scores and quality of life in patients with a history of mild, moderate, and severe TBI. The fitted lines indicate mean, and standard errors are from the linear mixed‐effects models covaried for age, education, and sex. The cognitive composite test results are shown as *t* score (50 is population mean, +/− 10 is one standard deviation greater or lower). The gray horizontal dashed lines indicate the mean for controls and are overlayed for clarity purposes. The trajectory of changes at individual level for cognitive composite and scores are shown in Figure S2 in supporting information. SWLS, Satisfaction with Life Scale.

Significant improvements over 5 years were seen in attention/processing speed and executive functioning composite scores across all TBI severities (Figure 4A,B). Both moderate and severe TBI patients showed significant improvements in delayed memory (Figure 4C), while severe TBI patients improved in language (Figure 4D). No significant changes were exhibited in the working memory domain over 5 years (Figure 4E). The trajectory of changes at individual participant level for cognitive composite scores are shown in Figure S2 in supporting information.

With respect to QoL, moderate TBI patients showed significant improvements in QoL as measured by the SWLS over time, while no significant changes were observed for those with a history of mild to moderate TBI (Figure 4F; Figure S2).

### 3.5 Cross‐sectional associations between serum biomarkers and brain volumes and cognition

**FIGURE 5:**
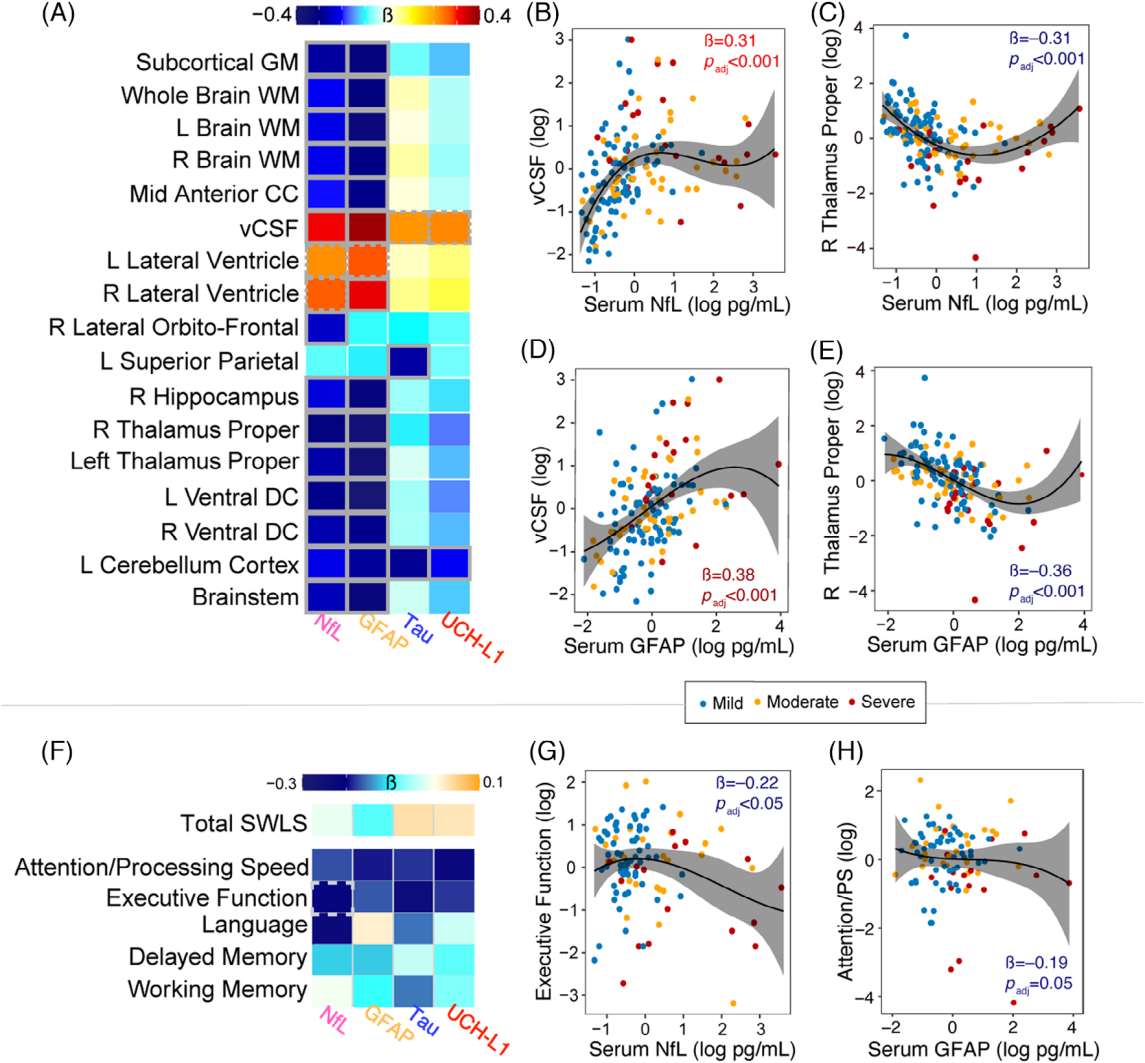
Cross‐sectional associations among serum biomarkers, brain volume, and cognitive assessments. A, Correlation between serum biomarkers and global and regional MRI‐measured brain volumes. All outcome measures were collected median 0.7 years, interquartile range 0.2–1.4 years after injury, but on the same day (± 1 day). In the heatmaps, the brain volumes and the serum biomarker concentrations were standardized for comparison purposes. Cooler colors indicate negative correlations, and hotter colors indicate positive. The correlations were assessed using linear models adjusted for age, sex, education, and time since most recent injury. Bold outlines, *P *< 0.01; dashed outlines *P *< 0.05, corrected for multiple comparison using Benjamini–Hochberg method. B–E, Examples of correlations summarized in the heatmap. F, Correlation between serum biomarkers and quality of life and cognitive composite scores. These associations were calculated in similar fashion as the other plots in this figure. The spline plot including the 95% confidence interval is shown for better depicting the direction of the associations. CC, corpus callosum; GM, gray matter, L, left; MRI, magnetic resonance imaging; R, right; SWLS, Satisfaction with Life Scale; vCSF, ventricular cerebrospinal fluid; WM, white matter.

Figure 5A shows the associations between global and regional brain volumes (measured at baseline) and concentrations of NfL, GFAP,[Fig alz13898-fig-0001], [Fig alz13898-fig-0002], [Fig alz13898-fig-0003], [Fig alz13898-fig-0004] tau, and UCH‐L1 (measured at baseline) in serum covaried for age, sex, and education, which also remained statistically significant after correcting for multiple comparison testing. Figures 5B–E show the individual patient data plotted for a few key brain regions summarized in Figure 5A. Increased serum NfL and GFAP were independently associated with lower GM and WM volumes. For GM, it included subcortical, frontal, temporal, and parietal volumes. For WM, it included WBWM and brainstem volumes. Also, increased serum NfL and GFAP were associated with increased ventricular CSF (vCSF) volumes (Figure 5A,E). Additionally, increased tau and UCH‐L1 were associated with lower regional volumes but the effect sizes and the number of associations that remained significant after correcting for multiple comparisons were small (Figure 5A). In summary, increased serum NfL and GFAP correlate with lower WBGM and WBWM volumes—essentially with the same brain regions that also showed progressive volume loss over time.

With respect to the serum biomarkers relationship to cognitive performance, increased concentrations of NfL were associated with lower executive function composite scores (*ß* = 0.21; Figures 5F–H). No other significant relationships between serum NfL or the other serum biomarkers and cognitive performance or QoL were observed after correcting for multiple comparison testing (Figure 5F).

### 3.6 Baseline serum biomarkers in relation to longitudinal changes in MRI brain volume

**FIGURE 6 alz13898-fig-0006:**
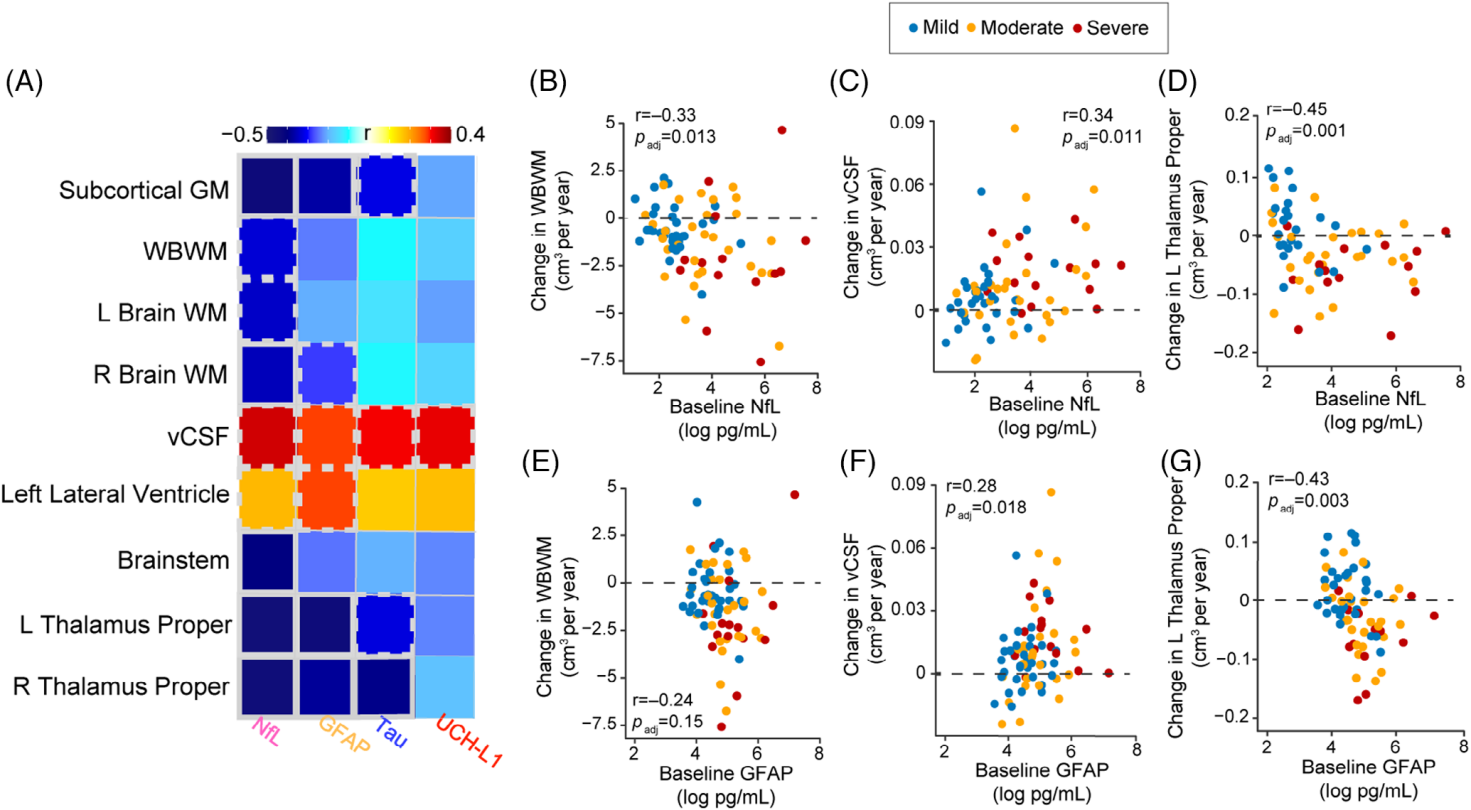
Serum NfL at baseline is associated with longitudinal changes in brain volumes. A–G, Concentrations of NfL, GFAP, tau, and UCH‐L1 measured at baseline predicting changes (in cm^3^ per year) in brain volume over time. B‐G, Examples of the correlations summarized in the heatmap. The changes in brain volumes were tested using linear mixed effects model, covaried for age, sex, and education. The association between the individual slopes from the linear mixed effects model and the baseline serum biomarker concentrations were tested using Spearman sign rank correlation (*r*). Only the associations that survived multiple comparisons are shown. The cooler colors in the heatmap indicate negative correlation and the hotter colors indicate positive. Bold outlines, *P *< 0.01; dashed outlines *P *< 0.05, Benjamini–Hochberg corrected. GFAP, glial fibrillary acidic protein; GM, gray matter, L, left; NfL, neurofilament light; R, right; vCSF, ventricular cerebrospinal fluid; WBWM, whole brain white matter; WM, white matter; UCH‐L1, ubiquitin carboxy‐terminal hydrolase‐L1.

Increased serum NfL and GFAP levels at baseline were significantly associated with greater longitudinal decline in GM, subcortical GM, and WBWM volumes (Figure 6A‐G). For GM, it included temporal, hippocampal, and thalamus proper volumes. For WM, it included CC and brainstem volumes. Additionally, higher serum NfL at baseline was associated with increased vCSF volume (Figure 6A). Increased baseline tau was associated with greater decline in left superior temporal, right thalamus proper, cerebellar cortex, and brainstem volumes (Figure 6A). Increased serum UCH‐L1 at baseline was associated with a faster rate of decline in volume in the thalamus proper and increased overall vCSF volume (Figure 6A). In summary, the associations between baseline serum NfL levels and changes in brain atrophy over 5 years were stronger than for the other measured biomarkers.

There were no significant associations between baseline levels of the serum biomarkers and rate of change in cognition and QoL after correcting for multiple comparisons (Figure S3 in supporting information).

### 3.7 Longitudinal changes in serum NfL are associated with changes in brain volume loss

The trajectory of changes at individual participant level for serum biomarkers are shown in Figure S4 in supporting information. Table 2 shows the correlations between longitudinal changes in serum NfL, GFAP, tau, and UCH‐L1 and longitudinal changes in brain volumes, cognition, and QoL over 5 years. Overall, the changes in serum NfL levels followed the changes in both global and regional brain volumes over time. Slower rate of decline in serum NfL was associated with faster rate of subcortical GM, WBWM, brainstem, CC, and thalamus proper volume loss (Table 2). Also, a slower rate of decline in serum NfL levels was associated with increased ventricular expansion (Table 2). In the left inferior temporal brain regions, a faster rate of decline in NfL and tau was associated with faster decline in brain volume (driven by a few outliers and reflects the challenge of quantify these inferior brain regions with MRI; Table 2). Changes in GFAP, tau, and UCH‐L1 were not significantly associated with regional volume changes after correcting for multiple comparisons; however, there were associations between increased tau, UCH‐L1, and vCSF and thalamus proper (Table 2).

**TABLE 2:** Associations among longitudinal changes in NfL, GFAP, tau, and UCH‐L1 with longitudinal changes in brain volume, cognitive outcome after traumatic brain injury.

	NfL		GFAP		Tau		UCH‐L1	
Outcome Measures	82		82		82		82	
*n*	*ρ* ^a^	*P* value^b^	*ρ* ^a^	*P* value^b^	*ρ* ^a^	*P* value^b^	*ρ* ^a^	*P* value^b^
**Brain volume**								
Subcortical GM	**0.44**	**<0.0001 (0.0010)**	0.19	0.09 (0.11)	0.12	0.26 (0.37)	−0.21	0.06 (0.07)
Whole brain WM	**0.33**	**<0.001 (<0.01)**	−0.03	0.80 (0.21)	0.18	0.10 (0.11)	−0.14	0.22 (0.33)
L Brain WM	**0.34**	**0.002 (<0.01)**	0.05	0.68 (0.79)	0.16	0.15 (0.26)	−0.19	0.10 (0.21)
R Brain WM	**0.34**	**0.003 (<0.01)**	0.006	0.96 (0.96)	0.16	0.17 (0.28)	−0.14	0.22 (0.33)
Brainstem	**0.40**	**0.003 (<0.01)**	0.08	0.46 (0.57)	0.24	0.03 (0.14)	−0.21	0.05 (0.16)
vCSF	−**0.30**	**0.006 (0.012)**	−0.09	0.40 (0.51)	−0.16	0.16 (0.27)	0.25	0.02 (0.11)
Central CC	**0.27**	**0.015 (<0.05)**	0.02	0.85 (0.85)	0.18	0.11 (0.21)	−0.21	0.06 (0.17)
R bankssts	−**0.30**	**0.006 (0.011)**	−0.18	0.11 (0.22)	−0.14	0.21 (0.32)	−0.01	0.90 (0.90)
L transverse temporal	−0.16	0.14 (0.20)	−004	0.70 (0.80)	−**0.37**	**0.009 (<0.01)**	−0.08	0.48 (0.60)
L inferior temporal	−**0.29**	**0.009 (0.012)**	−0.18	0.11 (0.21)	−**0.28**	**0.011 (<0.05)**	−0.002	0.99 (0.99)
L thalamus proper	**0.37**	**<0.001 (<0.01)**	0.28	0.011 (0.015)	0.06	0.62 (0.72)	−0.18	0.12 (0.21)
R thalamus proper	**0.38**	**<0.001 (<0.01)**	**0.30**	**0.005 (0.010)**	0.07	0.52 (0.61)	−0.18	0.11 (0.22)
**Cognitive composite score**								
Attention/processing speed	−0.10 (71)^c^	0.38 (0.71)	0.10 (71)^c^	0.40 (0.70)	0.02 (70)^c^	0.90 (0.97)	−0.005 (70)^c^	0.96 (0.99)
Executive function	−0.11 (71)	0.37 (0.74)	0.04 (71)	0.75 (0.91)	0.06 (70)	0.59 (0.79)	−0.003 (70)	0.98 (0.98)
Language	−0.11 (71)	0.34 (0.80)	−0.02 (71)	0.86 (0.96)	−0.04 (70)	0.80 (0.92)	−0.12 (70)	0.32 (0.82)
Delayed memory	−0.32 (71)	0.007 (0.09)	−0.14 (71)	0.23 (0.83)	−0.12 (70)	0.32 (0.89)	0.23 (70)	0.05 (0.38)
Working memory	−0.08 (72)	0.49 (0.72)	−0.12 (72)	0.30 (0.94)	−0.27 (71)	0.021 (0.20)	0.11 (71)	0.49 (0.72)
**Quality of life**								
Total SWLS	−0.04 (72)	0.72 (0.91)	0.09 (72)	0.42 (0.69)	−0.15 (71)	0.17 (0.69)	0.17 (71)	0.12 (0.66)

Abbreviations: bankssts, banks of the superior temporal sulcus; CC, corpus callosum; GFAP, glial fibrillary acidic protein; GM, gray matter; L, left; NfL, neurofilament light; R, right; SWLS, Satisfaction with Life Scale; UCH‐L1, ubiquitin carboxy‐terminal hydrolase‐L1; vCSF, ventricular cerebrospinal fluid; WM, white matter.

^a^ Correlations between slopes for serum biomarkers and slopes for brain volumes and cognitive composite outcomes. Slopes for serum biomarkers and other measures were estimated in separate linear mixed‐effects models (adjusted for age, sex, and education) and then correlated with each other using Spearman rank correlation. Only brain regions that remained statistically significant after adjusting for multiplicity testing are shown in the table.

^b^ *P* values within parentheses are corrected for multiple comparisons using the Benjamini–Hochberg method and P values that survived multiple comparisons are shown in bold.

^c^ Number of participants are shown within parenthesis with paired longitudinal blood biomarkers and cognitive data.

The correlations between changes in the serum biomarkers and any changes in cognitive composite scores did not remain significant after correcting for multiple testing (Table 2).

## 4 DISCUSSION

The main findings of this study are: (1) at 5 years post injury, patients with TBI had progressive rates of brain atrophy as reflected by decreases in GM, WM, and ventricular volume expansion compared to the baseline scan; (2) TAI is associated with the progression of brain atrophy; (3) in general, patients with TBI showed improvements in cognition and QoL over the same period; and (4) initial and longitudinal changes in serum NfL and GFAP measures independently predicted future brain volume changes.

Many studies suggest that TBI, mainly moderate to severe TBI, is associated with progressive brain atrophy.^6^, ^7^, ^13^, ^14^, ^15^, ^16^, ^17^, ^18^, ^39^, ^40^ Our study extends earlier work in a larger sample, includes TBI patients across the severity spectrum, and serial assessments up to 5 years post injury. Consistent with existing studies, we show that TBI of all severities is associated with smaller brain volumes compared to controls at mean 1 year after injury.^13^, ^14^, ^15^, ^16^, ^17^, ^18^ A recent study reported greater rate of atrophy in the WBGM and WBWM in a small sample of patients with moderate to severe TBI compared to controls at ≈ 5 years.^40^ Our study confirms these findings in a substantially larger cohort using multiple time points showing that brain atrophy after moderate to severe TBI is progressive and may continue for years after the initial trauma. The paucity of data on longitudinal changes for uninjured controls makes the findings about progressive atrophy in the MRI negative mild TBI cases preliminary and in need of confirmation. In addition, we observed differences in the spatial pattern of atrophy between those with mild TBI, and those with moderate to severe TBI. In patients with mild TBI, progressive atrophy was seen in the GM of temporal and parietal brain regions, while in those with moderate to severe TBI, atrophy was more pronounced in the subcortical GM and WM structures. The differences in the spatial pattern of atrophy between mild and moderate to severe TBI seen herein could be explained by the heterogeneity of the initial biomechanical loading conditions, which is beyond the scope of the current study. The average rates of global brain atrophy seen in this cohort is comparable to the recent study by Cole et al.;^1^ however, they are lower than other earlier studies that reported global atrophy ranging from 4% to 8.5%.^6^, ^7^, ^13^, ^14^ There are several reasons for higher rate of brain atrophy reported in the previous studies, including earlier assessments in the acute and subacute phase after TBI (which may reflect a faster rate of atrophy over the first 6–12 months after injury, and is potentially confounded by acute edema), high variability between scans, and small sample sizes. Last, multiple MRI assessments over 5 years provides a more robust approach and estimate of the progression of brain atrophy rather than MRIs at two time points.

TAI has been hypothesized to accelerate the rate of normal brain atrophy.^1^, ^6^, ^7^, ^13^, ^14^, ^15^, ^16^, ^17^, ^18^, ^19^, ^39^, ^40^ In support of this hypothesis, we found that the extent of DTI measures of TAI at baseline were associated with the rate of WBGM, subcortical GM, and WBWM volume loss. These findings are consistent with a recent study^41^ showing that TAI as measured by DTI FA is associated with the progression of brain atrophy in those with moderate to severe TBI. Here, we also extend these findings to patients with mild TBI and assessments over 5 years. It is important to acknowledge that we used CC as a surrogate marker of overall TAI, which is not a pathoanatomically specific injury subtype that is related to late atrophy. The exact mechanism of how TAI is associated with progressive neurodegeneration is not yet fully elucidated in humans. Wallerian degeneration of axons may play a role especially in the early phase, and also the failure of the glia cells to clear the myelin breakdown products which can be seen in WM tracts years after TBI.^42^ TAI is thought to cause impaired transport along the damaged axons and thereby promote hyperphosphorylation of tau and accumulation of amyloid beta, both of which are toxic, and also hallmarks of other progressive neurodegenerative diseases.^43^ Together, these findings suggest that WM microstructural disruptions are associated with and may contribute to accelerated age‐related brain atrophy.

Existing studies suggest that TBI causes long‐term functional and neuropsychological impairment.^20^, ^44^, ^45^ We performed detailed characterization of changes in cognition by constructing composite scores. Composite neuropsychological scores often outperform individual test results in terms of reliability and generalizability.^46^ We observed improvement over 5 years in the cognitive domains of attention/processing speed, executive function, language, and delayed and working memory as well as improvement in QoL. Although cognitive composite scores and QoL improved over time, patients with moderate to severe TBI still exhibited lower scores than mild TBI patients. There are no existing longitudinal studies that assessed cognitive performance after TBI in detail, hindering direct comparison. The improvement in cognitive performance seen herein is in stark contrast to the progressive brain atrophy we observed. There are several possible reasons for this, including the existence of functional compensation within brain networks, meaning atrophy could occur within a network without major impact on cognitive performance.^47^ As is evident by the findings in this study, the relationship between cognitive performance and neurodegeneration after TBI is complex, and suggests two distinct processes, in which there is an interaction between them early on, followed by a divergence in the years after injury. Because TBI is associated with increased risk of dementia decades after the injury, the post‐traumatic atrophy reported here may nonetheless be highly consequential.^48^, ^49^ Future studies with long‐term follow‐up (beyond 5 years) would be able to clarify the time course of post‐traumatic neurodegeneration and its relationship to cognitive impairment.

We have previously observed significant correlations between serum biomarkers (NfL, GFAP, tau, and UCH‐L1) and global[Fig alz13898-fig-0006] brain volumes cross‐sectionally in this cohort.^31^ Similar results were also observed by Graham et al. in patients with moderate to severe TBI, in whom initial serum NfL levels correlated with MRI‐measured neurodegeneration at 1 year.^39^ In the present study, paired serum biomarkers and MRIs allowed us to conduct detailed assessment of these biomarkers in relation to both global and regional volumes at baseline and longitudinally. We found that increased serum NfL and GFAP levels at baseline were associated with an accelerated rate of both global and regional brain atrophy; however, only longitudinal changes in serum NfL tracked the changes in both global and regional brain volumes over 5 years. Furthermore, tau and UCH‐L1 in serum demonstrated associations with regional brain volume changes, but these associations were weaker compared to those of NfL and GFAP. Similar findings for serum NfL have been seen in a recent study including eight patients with moderate to severe TBI who had serum NfL data and MRI scans at 8 months and ≈ 5 years later, where higher NfL levels at 8 months were associated with increased brain global atrophy at ≈ 5 years.^40^ Together, these findings suggest that serum NfL and GFAP show promise as a prognostic markers of progressive brain volume loss after TBI. The real‐world clinical utility of blood NfL and GFAP are yet to be tested in patients with TBI. Based on our experience of the clinical and research utility of blood NfL in multiple sclerosis, NfL and/or GFAP may be used for allocation of rehabilitative resources, and selection of participants for clinical trials of neuro‐restorative therapies, and monitoring the response to such therapies.^50^

Last, we observed correlations between serum NfL and executive functioning composite score. The relationship between serum NfL or other measured serum biomarkers and cognitive outcome measures has not been assessed over multiple time points and years previously, hindering direct comparison. Nevertheless, given the rapid rate of recovery seen here in all the outcome measures of interest, it seems plausible that the relationship between serum NfL as well as other serum biomarkers measured herein and cognitive outcomes would be weaker in the years after a TBI. Together, these findings suggest that serum NfL may have a potential role as prognostic markers months to years after a TBI.^51^

There are limitations to this study. First, we did not have longitudinal data available at all time points for the entire TBI cohort, which is a limitation of many longitudinal studies. Prior to the start of the study, we decided to account for this issue by using LME modeling. Second, the controls had fewer follow‐up MRI scan to precisely assess the age‐related rate of brain atrophy in healthy individuals. Nevertheless, the annualized rate of atrophy seen in our controls is similar to the previous study^1^ but higher than observed by Newcombe et al.^40^ Third, we have enrolled ≈ 56% mild TBI and ≈ 54% with moderate–severe TBI, based on the DoD/VA definition of TBI severity.^32^ Our proportion of mild cases is slightly lower relative to the previously published studies because we only included those who did not have conventional imaging abnormalities in the mild group, restricting direct comparison with prior mild TBI studies. However, the almost equal distribution between mild and moderate–severe TBI allowed for a better comparison of atrophy patterns across the severities. Fourth, the time from head trauma to enrollment was wide for the cross‐sectional analysis; however, a majority of the patients were enrolled ≈ 0.7 to 1 year after a TBI. Last, we observed higher analytical variations in tau and UCH‐L1, especially at lower concentrations, limiting the utility of these biomarker in subacute or chronic TBI.

To conclude, we found evidence of progressive brain volume loss months to years after a TBI. Spatial patterns of atrophy differ by injury severity and TAI is associated with the progression of brain atrophy. In contrast to the changes in brain volume, cognitive measures improved over time, suggesting two separate processes are taking place. Serum NfL and GFAP show promise as non‐invasive prognostic biomarkers of progressive neurodegeneration in TBI and may be useful for patient stratification and monitoring of treatment effects in trials of disease‐modifying therapies.

## CONFLICT OF INTEREST STATEMENT

The authors declare no conflicts of interest. Author disclosures are available in the supporting information.

## CONSENT STATEMENT

All human subjects provided informed consent.

## Supporting information

Supporting information

Supporting information

## Data Availability

The data supporting the findings may be available upon request.
