# Supplementary material for: Serum NfL and GFAP as biomarkers of progressive neurodegeneration in TBI
Source: Alzheimers Dement. 2024 May 28;20(7):4663–76. doi: 10.1002/alz.13898 (PMC11247683; doi:10.1002/alz.13898)
Supplement: Supplementary file 1 — Supporting information [file ALZ-20-4663-s002.docx]

**Supplementary Appendix**

**Serum NfL and GFAP as biomarkers of progressive neurodegeneration in TBI**

Pashtun Shahim^1-5^, MD, PhD, Dzung L. Pham^4,6^, PhD, Andre J. van der Merwe^1,4,5^, BSc, Brian Moore^1,4,5^, PA-C, MPH, Yi-Yu Chou^4,5^, MS, Sara M. Lippa^6,7^, PhD, Kimbra Kenney^6,7^, MD, PhD, Ramon Diaz-Arrastia^8^, MD, PhD, Leighton Chan^1^, MD, MPH

^1^Rehabilitation Medicine Department, National Institutes of Health (NIH) Clinical Center, Bethesda, MD, USA

^2^National Institutes of Neurological Disorders and Stroke, NIH, Bethesda, MD, USA

^3^Department of Neurology, MedStar Georgetown University Hospital, Washington DC, USA

^4^The Military Traumatic Brain Injury Initiative (MTBI2)

^5^The Henry M. Jackson Foundation for the Advancement of Military Medicine, Bethesda, MD, USA

^6^Uniformed Services University of the Health Sciences, Bethesda, MD, USA

^7^National Intrepid Center of Excellence, Walter Reed National Military Medical Center, Bethesda, MD, USA

^8^Department of Neurology, University of Pennsylvania Perelman School of Medicine, Philadelphia, PA, USA

**Materials and methods**

**Study design and participants**

A detailed study protocol and the inclusion and exclusion criteria can be found on ([ClinicalTrials.gov](http://clinicaltrials.gov/) Identifier: NCT01132898). In summary, the inclusion criteria for the TBI participants were: (*1*) male or female over 18 years of age; (*2*) clinical diagnosis of non-penetrating TBI, and (*3*) injury occurring less than one year before enrolment. Exclusion criteria included: (*1*) contraindications to MRI, including foreign metallic objects and non-compatible metallic devices, (*2*) medical or psychological instability such that the subject could not reasonably be expected to fulfil the study requirements and (*3*) pregnancy. The severity of TBI was based on the Department of Defence (DoD) and Veteran Affairs (VA) criteria [1] and clinical history. A patient was classified as a mild TBI if Glasgow Comas Scale (GCS) score in the emergency room was 13-15, there was no abnormality on brain CT or conventional clinical MRI, and if they experienced less than 30 minutes of loss of consciousness (LOC), less than 24 hours of post-traumatic amnesia (PTA) or alteration of consciousness (AOC). The moderate TBI criteria included those with GCS 9-12 or those with GCS 13-15 with any neuroimaging abnormalities, LOC > 30 minutes but less than 24 hours, AOC >24 hours, and PTA >24 hour but less than < 7 days. Severe TBIs were those with GCS 3-8, LOC ≥24 hours, or PTA ≥ 7 days.

Inclusion criteria for healthy controls were: (*1*) 18 years of age or older (*2*) good general medical and psychological health based on history and physical by licensed medical staff (*3*) no history of heavy alcohol use or substance abuse (*4*) no history of prior head injury, regardless of cause. Civilian participants were recruited via community outreach, study referral from acute TBI protocol [[ClinicalTrials.gov](http://clinicaltrials.gov/) Identifier: NCT01132937], and physician referral. Participants were enrolled between January 2011 and February 2020 at the National Institutes of Health (NIH) Clinical Center, Bethesda, MD, USA. The participants were also offered longitudinal blood, imaging, and outcome assessments at 30 (± 10 days), 90 (± 30 days), and 180-day (± 30 days), and at 1, 2, 3, 4, and 5 years (± 2 months).

**Image acquisition and processing**

High resolution structural MR and diffusion weighted images (DWIs) were acquired on a 3 tesla MR scanner (Siemens Biograph) with a 16-channel head coil in Radiology and Imaging Sciences at the NIH, Bethesda, MD, USA. Structural images were acquired on the same scanner as DWIs. In summary, the T_1_-weighted Magnetization Prepared Rapid Gradient Echo (MPRAGE) imaging included the following parameters: TR=2530 ms, TE=3.03 ms, flip angle=7 degrees, voxel size=1x1x1 mm, matrix size=256 x 256, and slices=176. We corrected the images for intensity non-uniformity using the N4ITK algorithm [2]. The images were de-skulled using MONSTR [3]. Anatomical brain segmentations (grey matter [GM], white matter [WM], and cerebrospinal fluid [CSF]) were performed via FreeSurfer (v.6.3.0 [http://surfer.nmr.mgh.harvard.edu](http://surfer.nmr.mgh.harvard.edu/)) analysis of the T_1_-weighted MPRAGE images. Volumetric measures were adjusted for total intracranial volume (ICV) using a previously described method whereby the imaging measure (y) is regressed on ICV (x) in the control group, and the adjusted imaging measure for all patients is subsequently computed as the residual value for the regression line [4]. Focal cerebral lesions were seen in seven patients; their T_1_-weighted images were segmented into lobes using FreeSurfer, and the structures within the affected lobes were excluded from the analysis.

DWIs were processed using the TORTOISE software for tensor estimation [5]. Images were pre-processed for motion correction and eddy current correction, with adjustments to the gradient table performed based on patient position. Distortions due to echo planar imaging susceptibility artefacts were corrected by first performing brain extraction on an anatomic T_2_-weighted SPACE acquisition (TR=3200 mm, TE=280 mms, flip angle=120 degrees, spatial resolution=0.98 x 0.98 x 1 mm, resampled to 0.49 x 0.49 x 1 mm). A rigid registration was performed that aligned the T_2_-weighted image to the b=0 image using the ANTS software package [6]. A deformable registration was performed within TORTOISE from the b=0 image to the T_2_-weighted image, and the resulting transformation was applied to each gradient direction. After distortion correction, non-linear least-squares tensor estimation was performed followed by computation of fractional anisotropy (FA), axial diffusivity (AD), radial diffusivity (RD), and mean diffusivity (MD). Measurements were averaged across 39 regions of interest, defined automatically using the DOTS tract segmentation algorithm [7].

**Neuropsychological assessments**

We constructed composites scores for five key cognitive domains, each comprised of the following neuropsychological tests: (*1*) Attention/Processing Speed: Wechsler Adult Intelligence Scale-Fourth Edition (WAIS-IV),[8] Coding and Symbol Search Scaled Scores, Trail Making Test A Heaton *t* score;[9] (*2*) Working Memory: WAIS-IV Digit Span and Arithmetic Scaled Scores; (*3*) Delayed Memory: California Verbal Learning Test-Second Edition (CVLT-II) [10] Short Delay Free Recall *z* score, and Long Delay Free Recall *z* score; (*4*) Language: Boston Diagnostic Aphasia Examination (BDAE) Complex Ideational Material (CIM), Boston Naming Test (BNT), and Animal Fluency Heaton *t* score; and (*5*) Executive Functioning: Booklet Category Test (BCT) Total Errors *t* score, Phonemic Verbal Fluency Heaton *t* score, and Trail Making Test B Heaton *t* score. All scores were converted to *t* scores and averaged to form the composite scores. In the case of missing data, the composite scores were computed for all participants having at least two scores in each domain. Participants were also administered at least one performance validity test (PVT): The Medical Symptom Validity Test (MSVT);[11] Test of Memory Malingering (TOMM),[12] or the WAIS-IV Reliable Digit Span (RDS). To ensure poor effort was not driving reduced cognitive scores, failure of any of these three measures resulted in exclusion from neuropsychological analyses. For the MSVT and TOMM, failure was defined by cut-offs in the manual. For RDS, failure was defined as any score less than seven.[13]

In addition to cognitive domains, we assessed QoL, using the Satisfaction with Life Scale (SWLS), respectively.[14]

**Blood handling and processing**

Blood samples were collected by venepuncture into gel-separator tubes for serum and centrifuged within 20-60 minutes. Serum samples were aliquoted and stored at –80 °C pending biochemical analysis. Serum NfL, GFAP, tau, and UCH-L1 concentrations were measured using the Neurology 4-plex assay kit (Quanterix Corporation, Lexington, MA, USA) on a single molecule array HD-1 Analyzer (Quanterix Corporation, Lexington, MA, USA). The average coefficient of variation of measurement of NfL, GFAP, tau, and UCH-L1 were 4%, 3%, 33%, and 30%, respectively. All samples were processed and analysed at NIH, Bethesda, MD, USA, using the same batch of reagents by certified laboratory technicians blinded to clinical information.

| **Supplementary table 1. Demographic characteristics of the controls with longitudinal scans** | |
| --- | --- |
| n | 12 |
| Age, median (IQR) | 47 (42-53) |
| Sex, female/male | 7/5 |
| Race, no. (%) |  |
| White | 9 (75) |
| African American | 2 (17) |
| Multiple races | 1 (8) |
| Years of education, mean ± SD | 17±1.6 |


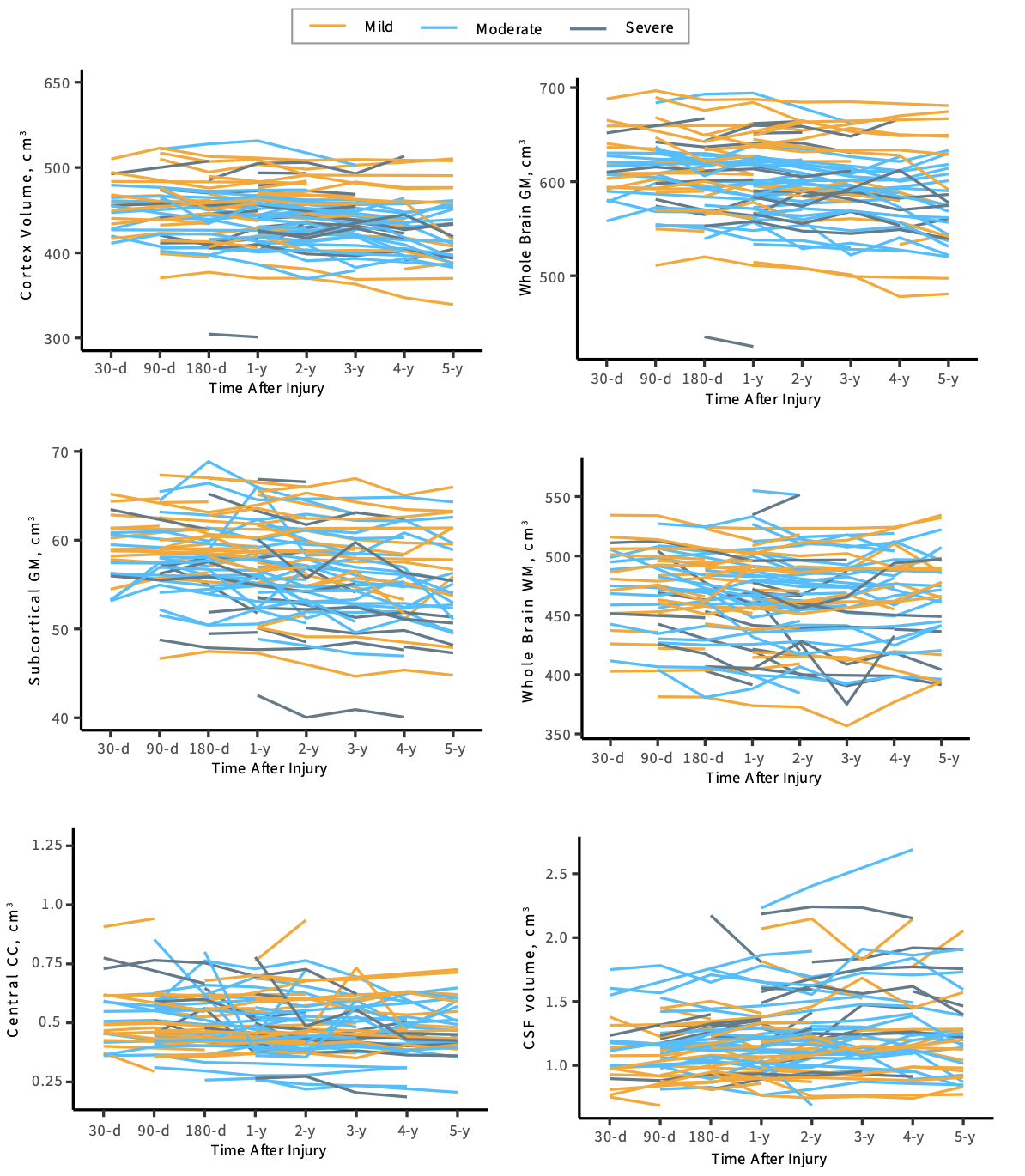


**Supplementary Fig 1. The trajectory of brain volume changes at individual participant level for few key brain regions over 5 years**. Each line represents an individual patient. Patients underwent MRI assessment at 30 (n=29), 90 (n=45), and 180 (n=55) days, and 1 (n=82), 2 (n=58), 3 (n=45), 4 (n=39), and 5 (n=33) years after injury. *Abbreviations*: GM = gray matter; WM = white matter; CC = corpus callosum; CSF = cerebrospinal fluid.


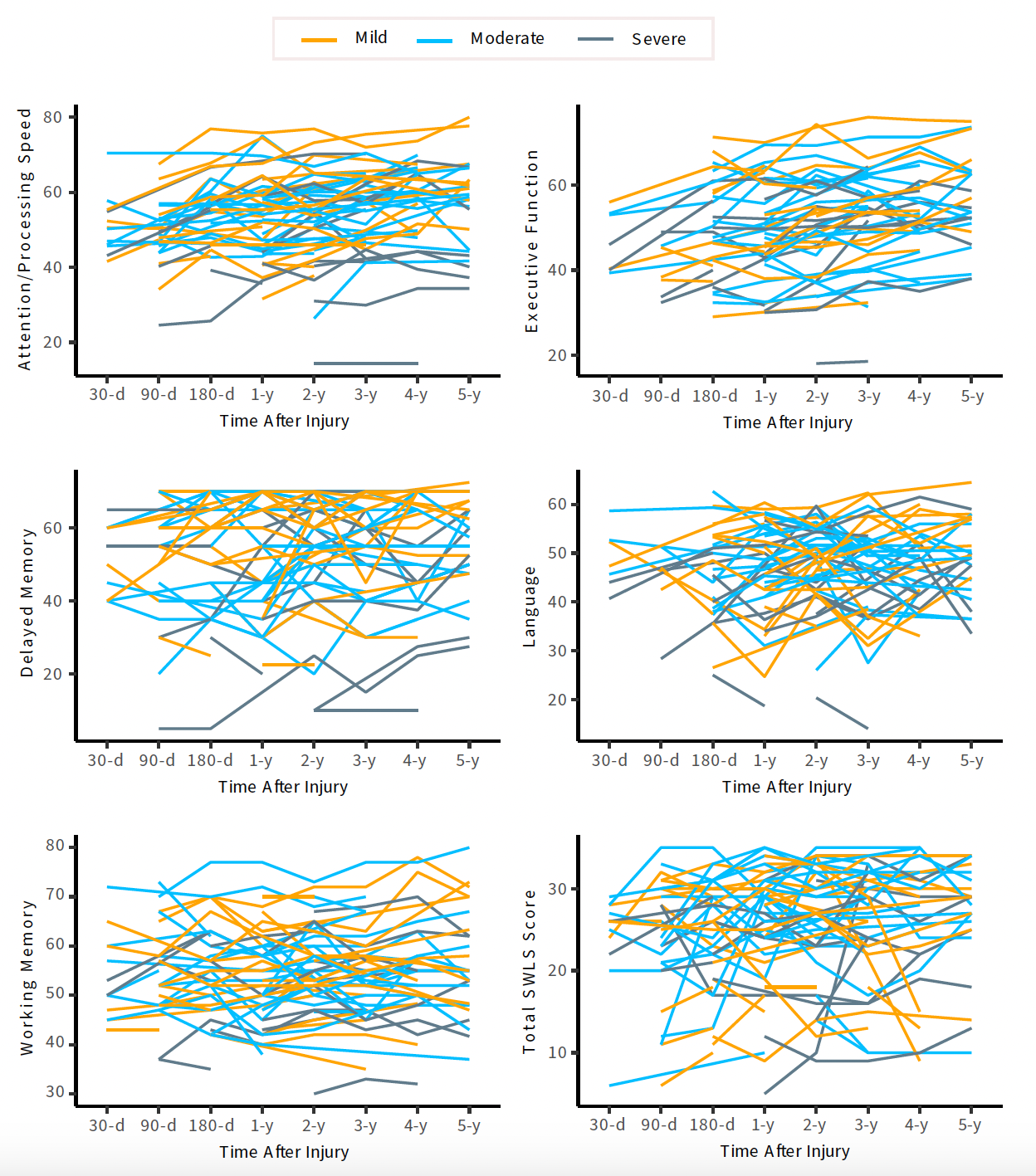


**Supplementary Fig 2. The trajectory of changes at individual level for cognitive composite scores and quality of life over 5 years.** Each line represents an individual patient. Patients underwent cognitive assessments at 30 (n=22), 90 (n=36), and 180 (n=47) days, and 1 (n=71), 2 (n=51), 3 (n=41), 4 (n=36), and 5 (n=30) years after injury. *Abbreviation*: SWLS = Satisfaction with Life Scale.


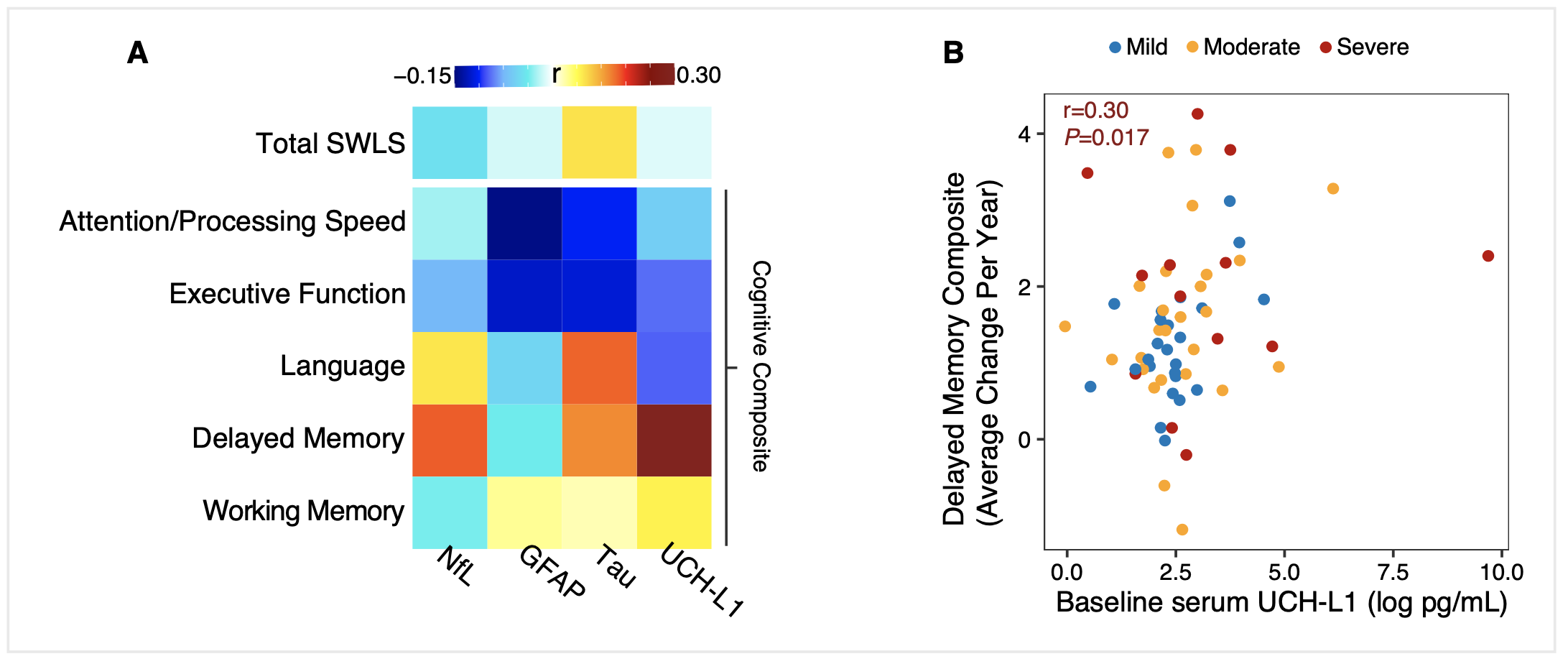


**Supplementary Fig 3. Serum biomarkers concentration at baseline in relation to changes in functional and neuropsychological outcomes over time.**

Plots A shows the concentrations of NfL, GFAP, tau, and UCH-L1 measured at baseline in relation to changes in functional outcome, quality of life, and cognitive performance over time. The cooler colours in the heatmap indicate positive correlation and the hotter colours indicate negative. The changes in functional and neuropsychological outcome measures over time were tested using linear-mixed effects model, covaried for age, sex, and education. The association between the individual slope and the baseline biomarker concentrations were tested using Spearman rank correlation (r), followed by Benjamini-Hochberg correction. The cognitive composite test results are shown as *t* score (50 is population mean, +/–10 is one standard deviation greater or lower). Plot B shows an example of the correlations shown in the heatmap (plot A). *Abbreviations*: NfL = neurofilament light; GFAP = glial fibrillary acidic protein; UCH-L1 = ubiquitin carboxy-terminal hydrolase-L1; GOS-E = Glasgow Outcome Scale-Extended; SWLS = Satisfaction with Life Scale.


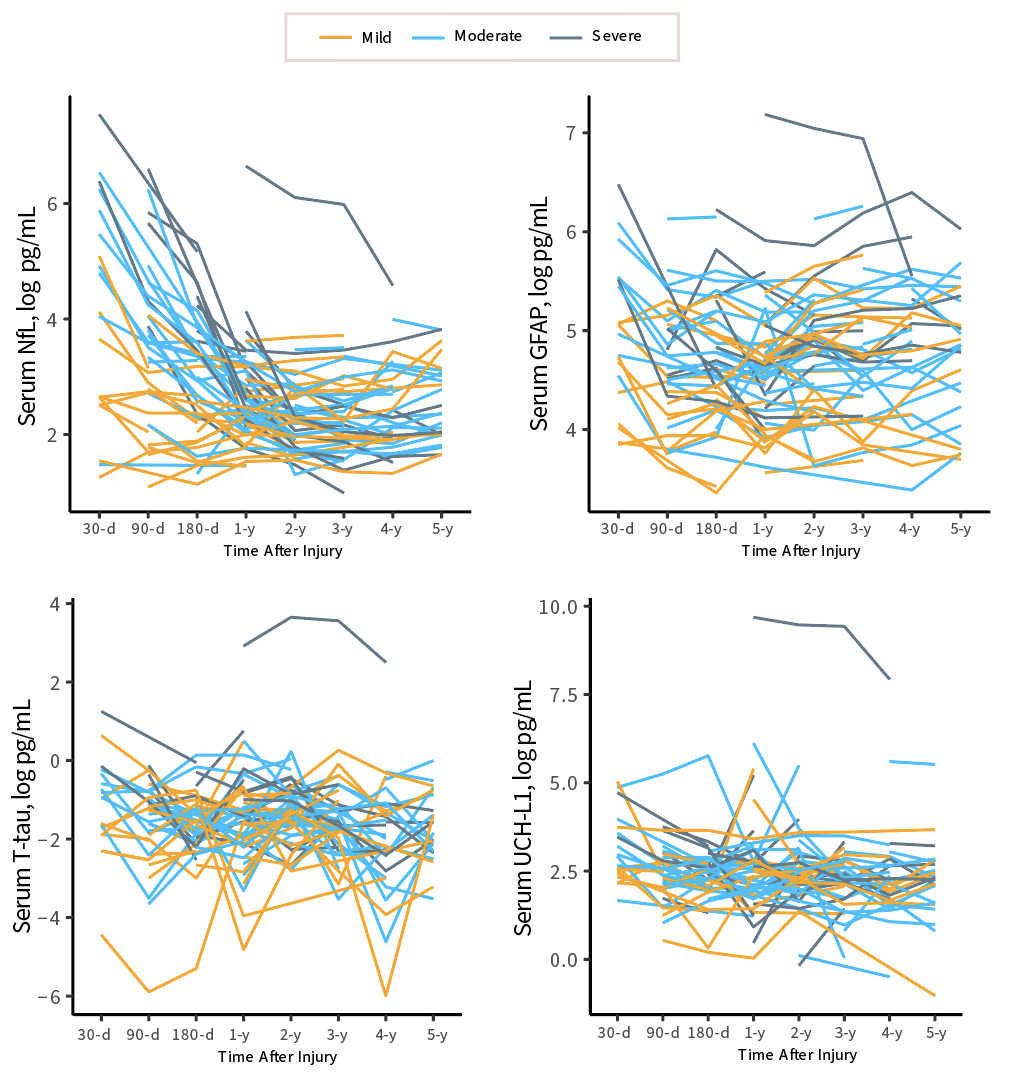


**Supplementary Fig 4. The trajectory of changes at individual level for serum neuronal injury biomarkers over five years.** Each line represents an individual patient. Patients underwent blood assessments at 30 (n=26), 90 (n=41), and 180 (n=51) days, and 1 (n=74), 2 (n=51), 3 (n=40), 4 (n=32), and 5 (n=24) years after injury. *Abbreviations*: NfL = neurofilament light; GFAP = glial fibrillary acidic protein; UCH-L1 = ubiquitin carboxy-terminal hydrolase-L1.

**References:**

[1] Management of Concussion/m TBIWG. VA/DoD Clinical Practice Guideline for Management of Concussion/Mild Traumatic Brain Injury. J Rehabil Res Dev. 2009;46:CP1-68.

[2] Tustison NJ, Avants BB, Cook PA, Zheng Y, Egan A, Yushkevich PA, Gee JC. N4ITK: improved N3 bias correction. IEEE Trans Med Imaging. 2010;29:1310-20.

[3] Roy S, Butman JA, Pham DL, Alzheimers Disease Neuroimaging I. Robust skull stripping using multiple MR image contrasts insensitive to pathology. Neuroimage. 2017;146:132-47.

[4] Voevodskaya O, Simmons A, Nordenskjold R, Kullberg J, Ahlstrom H, Lind L, et al. The effects of intracranial volume adjustment approaches on multiple regional MRI volumes in healthy aging and Alzheimer's disease. Front Aging Neurosci. 2014;6:264.

[5] C. Pierpaoli LW, M. O. Irfanoglu, A. Barnett, P. Basser, L-C. Chang, C. Koay, S. Pajevic, G. Rohde, J. Sarlls, and M. Wu. TORTOISE: an integrated software package for processing of diffusion MRI data. ISMRM 18th annual meeting, Stockholm, Sweden, 20102010.

[6] Avants BB, Tustison NJ, Song G, Cook PA, Klein A, Gee JC. A reproducible evaluation of ANTs similarity metric performance in brain image registration. Neuroimage. 2011;54:2033-44.

[7] Bazin PL, Ye C, Bogovic JA, Shiee N, Reich DS, Prince JL, Pham DL. Direct segmentation of the major white matter tracts in diffusion tensor images. Neuroimage. 2011;58:458-68.

[8] Wechsler D. Wechsler Adult Intelligence Scale-Fourth Edition: Technical and interpretive manual. San Antonio, TX: Pearson; 2008.

[9] Heaton RK, Miller SW, Taylor MJ, Grant I. Revised Comprehensive Norms for an Expanded Halstead Reitan Battery: Demographically Adjusted Neuropsychological Nroms for African American and Caucasian Adults. Lutz, FL: Psychological Assessment Resources, Inc; 2004.

[10] Delis DC, Kramer JH, Kaplan E, Ober BA. California Verbal Learning Test. 2 ed. San Antonio: Psychological Corporation; 2000.

[11] Green P. Green's Medical Symptom Validity Test (MSVT) for Microsoft Windows: User's manual. Edmonton, Canada: Green's Publishing; 2004.

[12] Tombaugh TN. TOMM: Test of Memory Malingering. Tonawanda, NY: Multi-Health Systems; 1996.

[13] Schroeder RW, Twumasi-Ankrah P, Baade LE, Marshall PS. Reliable Digit Span: a systematic review and cross-validation study. Assessment. 2012;19:21-30.

[14] Pavot W, Diener E, Colvin CR, Sandvik E. Further validation of the Satisfaction with Life Scale: evidence for the cross-method convergence of well-being measures. J Pers Assess. 1991;57:149-61.
